# Clinical and Therapeutic Features of Pulmonary Nontuberculous Mycobacterial Disease, Rio de Janeiro, Brazil

**DOI:** 10.3201/eid1903.120735

**Published:** 2013-03

**Authors:** Karla Gripp Couto de Mello, Fernanda C. Queiroz Mello, Liamar Borga, Valeria Rolla, Rafael S. Duarte, Elizabeth P. Sampaio, Steven M. Holland, D. Rebecca Prevots, Margareth P. Dalcolmo

**Affiliations:** Author affiliations: National School of Public Heath, Oswaldo Cruz Foundation, Rio de Janeiro, Brazil (K.G. Couto de Mello, L. Borga, M.P. Dalcolmo);; School of Medicine, Federal University of Rio de Janeiro, Rio de Janeiro (F.C. Queiroz Mello);; Oswaldo Cruz Institute, Rio de Janeiro (V. Rolla, E.P. Sampaio);; Federal University of Rio de Janeiro (R.S. Duarte);; National Institutes of Health, Bethesda, Maryland, USA (S.M. Holland, D.R. Prevots)

**Keywords:** mycobacteria, atypical, nontuberculous mycobacteria, tuberculosis and other mycobacteria, bacteria, Brazil

## Medscape CME Activity

Medscape, LLC is pleased to provide online continuing medical education (CME) for this journal article, allowing clinicians the opportunity to earn CME credit. 

This activity has been planned and implemented in accordance with the Essential Areas and policies of the Accreditation Council for Continuing Medical Education through the joint sponsorship of Medscape, LLC and Emerging Infectious Diseases. Medscape, LLC is accredited by the ACCME to provide continuing medical education for physicians.

Medscape, LLC designates this Journal-based CME activity for a maximum of 1 *AMA PRA Category 1 Credit(s)*^TM^. Physicians should claim only the credit commensurate with the extent of their participation in the activity.

All other clinicians completing this activity will be issued a certificate of participation. To participate in this journal CME activity: (1) review the learning objectives and author disclosures; (2) study the education content; (3) take the post-test with a 70% minimum passing score and complete the evaluation at www.medscape.org/journal/eid; (4) view/print certificate.

**Release date: February 22, 2013; Expiration date: February 22, 2014**

## Learning Objectives

Upon completion of this activity, participants will be able to:

- Analyze the epidemiology of pulmonary nontuberculous mycobacterial (PNTM) disease
- Evaluate the clinical presentation of PNTM disease
- Distinguish mycobacteria associated with the most cases of PNTM disease in the current study
- Distinguish the mycobacterium species associated with the lowest cure rates of PNTM in the current study.

## CME Editor

**Carol E. Snarey, MA, **Technical Writer/Editor, Emerging Infectious Diseases. *Disclosure: Carol E. Snarey, MA, has disclosed no relevant financial relationships.*

## CME Author

**Charles P. Vega, MD, **Health Sciences Clinical Professor; Residency Director, Department of Family Medicine, University of California, Irvine. *Disclosure: Charles P. Vega, MD, has disclosed no relevant financial relationships.*

## Authors

*Disclosures: Karla Mello, Fernanda Mello, Liamar Borga, Rafael Duarte, Elizabeth Sampaio, Steven Holland, Rebecca Prevots, and Margareth Dalcolmo have disclosed no relevant financial relationships. Valeria Rolla has disclosed the following relevant financial relationships: served as an advisor or consultant for Janssen-Cilag; received grants for clinical research from Janssen-Cilag.*
