# Supplementary material for: Clinical and Therapeutic Features of Pulmonary Nontuberculous Mycobacterial Disease, Rio de Janeiro, Brazil
Source: Emerg Infect Dis. 2013 Mar;19(3):393–9. doi: 10.3201/eid1903.120735 (PMC3647650; doi:10.3201/eid1903.120735)
Supplement: Related texts

# Research article: Clinical and Therapeutic Features of Pulmonary Nontuberculous Mycobacterial Disease, Rio de Janeiro, Brazil

**Authors:** Karla Gripp Couto de Mello, Fernanda C. Queiroz Mello, Liamar Borga, Valeria Rolla, Rafael S. Duarte, Elizabeth P. Sampaio, Steven M. Holland, D. Rebecca Prevots, Margareth P. Dalcolmo

**Affiliations:** Author affiliations: National School of Public Heath, Oswaldo Cruz Foundation, Rio de Janeiro, Brazil (K.G. Couto de Mello, L. Borga, M.P. Dalcolmo);; School of Medicine, Federal University of Rio de Janeiro, Rio de Janeiro (F.C. Queiroz Mello);; Oswaldo Cruz Institute, Rio de Janeiro (V. Rolla, E.P. Sampaio);; Federal University of Rio de Janeiro (R.S. Duarte);; National Institutes of Health, Bethesda, Maryland, USA (S.M. Holland, D.R. Prevots)

## Abstract

To identify clinical and therapeutic features of pulmonary nontuberculous mycobacterial (PNTM) disease, we conducted a retrospective analysis of patients referred to the Brazilian reference center, Oswaldo Cruz Foundation, Rio de Janeiro, Brazil, who received a diagnosis of PNTM during 1993–2011 with at least 1 respiratory culture positive for NTM. Associated conditions included bronchiectasis (21.8%), chronic obstructive pulmonary disease (20.7%), cardiovascular disease (15.5%), AIDS (9.8%), diabetes (9.8%), and hepatitis C (4.6%).Two patients had Hansen disease; 1 had Marfan syndrome. Four mycobacterial species comprised 85.6% of NTM infections: *Mycobacterium kansasii,* 59 cases (33.9%); *M. avium* complex, 53 (30.4%); *M. abscessus*, 23 (13.2%); and *M. fortuitum,* 14 (8.0%). A total of 42 (24.1%) cases were associated with rapidly growing mycobacteria. In countries with a high prevalence of tuberculosis, PNTM is likely misdiagnosed as tuberculosis, thus showing the need for improved capacity to diagnose mycobacterial disease as well as greater awareness of PNTM disease prevalence.

Pulmonary disease caused by nontuberculous mycobacteria (PNTM) frequently causes sickness and death. These bacteria are found in water sources and soil and are particularly concentrated in biofilms (*1**,**2*). Certain clinical conditions are known to be associated with an increased risk of PNTM, particularly immunosuppressive conditions and structural changes in the lung, such as those associated with chronic obstructive pulmonary disease (COPD), bronchiectasis, sequelae from prior pulmonary tuberculosis (TB), and cystic fibrosis (*3*).

Recent studies have documented the emergence of NTM lung diseases in industrialized countries, such as the United States (*4*,*5*). The current prevalence estimated for PNTM is ≈6 cases/100,000 population, with the highest prevalence in persons >50 years of age (*4*–*6*). In many developing countries with a high prevalence of TB, the prevalence of PNTM among immunocompetent persons remains unknown, largely because of the lack of routine culture and species identification from samples of persons with suspected cases. In Brazil in 2010, 70,601 cases of TB were reported, indicating a prevalence of 38 cases/100,000 population. In the same year, 4,500 deaths from TB were reported. In 2011, TB was the third leading cause of death (from infectious diseases) and the first among AIDS patients, according to surveillance data from Brazil’s National TB Control Program (*7*). However, because TB is routinely diagnosed presumptively, solely on the basis of identifying acid-fast bacilli (AFB) from sputum samples, an unknown proportion of patients may in fact be infected with NTM. Therefore, the true prevalence of NTM in Brazil remains unknown.

In Brazil, the Professsor Helio Fraga Reference Center (CRPHF) has served as a reference center for multidrug-resistant TB (MDR TB) and NTM since 1993. From 1993 to 2011, 5,638 cases of MDR TB were reported in Brazil, 1,894 of them in Rio de Janeiro; 1,595 of these patients were treated at CRPHF (*8*).

Prior studies have described some of the features of clinical isolates from PNTM patients (*9*–*14*), and others have described the clinical features for small populations infected with NTM clinical isolates in Brazil (*15*,*16*). To more fully describe the emerging prevalence and associated conditions of PNTM in a large urban population in Brazil, we present results of a large and detailed review of PNTM case-patients with PNTM who were treated at CRPHF.

## Materials and Methods

### Referral Population 

CRPHF is a national reference center for the diagnosis and treatment of MDR TB, and it also functions as a local reference center for treatment of PNTM case-patients from Rio de Janeiro through an outpatient unit. In Brazil, mycobacterial cultures are only performed for specific groups: 1) patients newly diagnosed with TB who remain positive for AFB positive in the second month of treatment; 2) patients who have a history of prior treatment for TB and are newly AFB positive; 3) patients who are contacts of persons with drug-resistant TB; and 4) patients who are part of specific population groups, including health professionals, the homeless, prisoners, indigenous populations, and HIV-positive persons (*7*). Thus, patients are referred to CRPHF from public and private healthcare facilities for mycobacterial species identification when NTM or MDR TB is suspected, typically because the patients remain AFB positive and do not improve clinically while receiving TB treatment, or when 1 sample culture is positive for NTM with no species identified. Radiographic evaluations are performed at baseline and at follow-up visits through x-ray films or, more recently, computed tomographic scans.

### Study Population and Data Collection

The study population comprised patients from the state of Rio de Janeiro who were referred to CRPHF for further evaluation, for either PNTM or MDR TB. Patients included in this analysis had at least 1 respiratory isolate identified as NTM, were residents in the state of Rio de Janeiro, and were referred to CRPHF during January 1993–January 2011. Only patients with NTM isolated from respiratory specimens were included. Demographic, clinical, and epidemiologic information was collected from patient clinical records. Information included co-existing conditions, smoking history, and results of radiographic imaging. With respect to microbiologic information, data included treatment duration for NTM disease, month of the first negative sputum culture, and treatment outcome. Additional information included the number of prior episodes of TB and prior treatment for TB, including information about those who had been treated for TB for at least 6 months before the diagnosis of NTM disease. Microbiologic confirmation with sputum culture and species identification were not able to be carried out for samples from patients’ prior TB episodes.

### Treatment and Assessment of Microbiologic Response 

After a patient’s diagnosis at CRPHF, sputum samples are collected monthly or bimonthly until treatment is completed. Subsequently, sputum samples are collected every 3 months in the first 12 months following treatment, and then every 6 months for at least 2 years following treatment. Recommended treatment depends on species, and generally follows American Thoracic Society (ATS) guidelines (*3*), with a course of 12–18 months for slow-growing mycobacteria, such as *M. kansasii* or *M. avium* complex (MAC), and longer courses for rapidly growing mycobacteria. The definitions for classification of treatment response were as follows: 1) cure was indicated by at least 3 consecutive respiratory specimens negative for NTM during 12 consecutive months; 2) treatment failure was indicated by at least 2 positive cultures at the end of 12 months of treatment; 3) relapse was indicated by cultures positive for NTM >30 days after a prior cure; and 4) death meant death from any cause during treatment, regardless of whether death was confirmed as associated with NTM disease.

### Laboratory Methods

Before 2004, species identification for *M. kansasii*, MAC, *M. abscessus,* and *M. fortuitum* was done through biochemical tests. Subsequently, the *hsp65* PCR restriction analysis (PRA) method was used for species identification

### Data Analysis

To assess association of species and cavitary disease with culture conversion, we used Epi Info version 3.5.3 (wwwn.cdc.gov/epiinfo/) and SAS version 9.2 (SAS Institute Inc., Cary, NC, USA). The significance of associations was assessed by using χ^2^ with p<0.05. We limited this analysis to those patients who met the ATS criteria (>1 NTM-positive sputum sample or 1 sample from bronchoalveolar lavage or lung biopsy specimens).

## Results

### Demographic and Clinical Features of Patients with NTM Lung Disease

We identified a total of 174 patients in our study population; 108 (62.1%) were male. The median age was 55 years (range 24–86 years). Smoking history was available for 153 patients, of whom 95 (62.1%) reported past or current smoking. The most frequent symptoms were respiratory (60.9%), but 30.5% of the total patients had both respiratory and systemic symptoms, including cough, dyspnea, hemoptysis, thoracic pain, fever, weight loss, and night sweats (Table 1). Overall, 101 (58.0%) patients reported prior treatment for TB, based only on a positive AFB smear (without microbiologic confirmation for *M. tuberculosis*), of whom 80 (79%) were referred while being treated empirically for TB for up to 6 months before the diagnosis of NTM infection. Overall, 127 (72.9%) patients met the ATS criteria for NTM disease (Table 1). The number of PNTM cases identified from 1993 to 2005 ranged from 5 to 7 cases per year. However, beginning in 2006, the number of identified cases reached ≈20, and it has remained at 20–40 cases per year since then (Figure).

**Table 1:** Demographic and clinical features for patients with PNTM, Rio de Janeiro, Brazil, 1993– 2011*

Characteristic	No. (%) patients
Male sex	108 (62.1)
Age, median y (range)	55 (24–86)†
Past or current smoker‡	95 (62.1)
Clinical findings	
Respiratory symptoms only	106 (60.9)
Fever and weight loss	15 (8.6)
Respiratory and systemic symptoms (fever, weight loss)	53 (30.5)
Previous TB treatment	
Treated previously for TB	101 (58)
Referred while being treated for TB	80 (79)
Radiology results (lesion type)	
Cavitary disease	118 (67.8)
Fibronodular	56 (32.2)
Median duration of treatment, mo (range)	19 (2–36)†
ATS microbiologic criteria	
1 positive sputum culture	47 (27)
>2 positive sputum cultures	101 1 (58)
Bronchoalveolar lavage	24 (13.8)
Lung biopsy	2 (1.1))

*PNTM, pulmonary nontuberculous mycobacterial disease; TB, tuberculosis; ATS, American Thoracic Society. †Data are as indicated in the left column. ‡Smoking data were missing for 21 patients.

**Figure:** Number of nontuberculous mycobacterial pulmonary disease cases per year, Professor Helio Fraga Reference Center, Rio de Janeiro, Brazil, January 1993–January 2011.

With respect to coexisting conditions, 59 (33.9%) patients had no identified coexisting conditions. The most frequently identified conditions were bronchiectasis (21.8%), COPD (20.7%), and cardiovascular disease (15.5%). A lower proportion of patients were identified with AIDS (9.8%), diabetes (9.8%), and hepatitis C (4.6%). All patients with bronchiectasis had this condition identified through computed tomographic scans. Two patients had Hansen disease, 1 of whom also had hepatitis C. One patient had Marfan syndrome and no other reported coexisting conditions (Table 2). Patients may have had >1 coexisting condition.

**Table 2:** Coexisting medical conditions for174 patients with PNTM, Brazil, 1993–2011*

Coexisting condition†	No. (%) patients
None	59 (33.9)
Bronchiectasis	38 (21.8)
Chronic obstructive pulmonary disease	36 (20.7)
Cardiovascular disease	27 (15.5)
AIDS	17 (9.8)
Diabetes	17 (9.8)
Asthma	8 (4.6)
Hepatitis C	8 (4.6)
Cancer, excluding lung cancer	6 (3.4)
Gastroesophageal reflux	6 (3.4)
Rheumatioid arthritis	3 (1.7)
Nonviral cirrhosis	2 (1.1)
Hansen disease	2 (1.1)
Lupus	2 (1.1)
Anemia falciforme	1 (0.57)
Silicosis	1 (0.57)
Marfan syndrome	1 (0.57)
Kidney transplantation and immunosupression	1 (0.57)
Mitral valve prolapse	1 (0.57)

*PNTM, pulmonary nontuberculous mycobacterial disease. †Patients may have exhibited >1 coexisting condition.

Patients without prior treatment for TB were not significantly different from those with prior treatment with respect to age, sex, infecting species, or clinical features. Among the 73 patients with no prior TB treatment, 47 (64.4%) were male with a mean age of 54 years. In this group, 43 (58.9%) showed cavitary lesions, of which 29 (67.4%) were bilateral.

### Species Identification and Response to Treatment

Four species comprised 85.6% of all species identified: *M. kansasii*, 33.9%, MAC, 30.4%, *M. abscessus*, 13.2%, and *M. fortuitum*, 8.0%. Overall, 42 (24.1%) patients had an isolate identified as a rapidly growing mycobacterium (*M. fortuitum, M. abscessus, M. peregrinum, M. massiliense*) (Table 3). The proportion of species identified through PRA was as follows: *M. kansasii,* 69.5%; MAC, 62.3%; *M. abscessus*, 55.5%; *M. fortuitum*, 57.1%; all other species, 100%. The species distribution in the group of patients with prior treatment for TB was similar to the overall distribution, with 37.3% infected with *M. kansasii* and 26.6% infected with MAC. The species distribution in the group of patients with bronchiectasis also showed a similar distribution: *M. kansasii* was the most frequent (31.5%), followed by MAC (31.5%), *M. abscessus* (10.5%), and *M. massiliense* (5.2%). Infection with uncommon species, such as *M. simiae, M. lentiflavum*, *M. celatum,* and *M. szulgai,* all occurred in this group.

**Table 3:** *Mycobacterium* species infecting 174 patients with PNTM, Brazil, 1993–2011

Species	No. (%) patients infected
*M. kansasii*	59 (33.9)
*Mycobacterium avium* complex	53 (30.4)
*M. abscessus*	23 (13.2)
*M. fortuitum*	14 (8.0)
*M. massiliense*	3 (1,7)
*M. peregrinum*	2 (1.1)
*M. asiaticun*	1 (0.57)
*M. simae*	1 (0.57)
*M. lentiflavum*	1 (0.57)
*M. szulgai*	1 (0.57)
*M. celatum*	1 (0.57)
*M. terrae*	1 (0.57)
Not identified	14 (8.0)

*PNTM, pulmonary nontuberculous mycobacterial disease.

Treatment outcome varied significantly by infecting species, with the highest cure rate (71.4%) observed among patients infected with *M. kansasii*, followed by those infected with MAC (57.8%), and the lowest cure rate (25.0%) was observed in patients infected with *M. abscessus* (Table 4). Although the majority of patients had cavitary disease, among those infected with *M. kansasii,* the outcome of disease was similar for those with cavitary and noncavitary lesions (72.7% and 67.0%, respectively); among those infected with MAC or *M. abscessus*, the cure rates appeared somewhat higher for those with noncavitary disease, although this effect was not significant (Table 5).

**Table 4:** Cure rates by species infecting patients with ATS-defined PNTM, Brazil, 1993–2011*

Species†	No. (%) patients cured	Total no.
*Mycobacterium kansasii*	30 (71.4)	42
*M. avian* complex	26 (57.8)	45
*M. abscessus*	6 (25.0)	24
*M. fortuitum*	5 (45.4)	11
Other	2 (40.0)	5
Total	69 (54.3)	127


*ATS, American Thoracic Society; PNTM, pulmonary nontuberculous mycobacterial disease. Patients without identified species were excluded from this analysis.

**Table 5:** Association of cavitary disease with cure rates of patients with ATS-defined PNTM, Brazil, 1993–2011*

Species	No. (%) patients		Relative risk (95% CI)
	With noncavitary disease	With cavitary disease	
*Mycobacterium kansasii*			
Cure	6 (67)	24 (72.7)	0.92 (0.55–1.5)
Total	9	33	
*M. avium* complex			
Cure	12 (85.7)	14 (45.2)	1.9 (1.2–3.0)
Total	14	31	
*M. abscessus*			
Cure	3 (42.9)	3 (17.7)	2.4 (0.64–9.2)
Total	7	14	

*ATS, American Thoracic Society; PNTM, pulmonary nontuberculous mycobacterial disease.

Treatment regimens by infecting species are shown in Table 6. Of the patients with MAC infection, 54.7% received combination antimicrobial drug therapy, consisting of rifampin, ethambutol, and clarithromycin; 52.0% of patients infected with *M. kansasii* underwent regimens containing rifampin, ethambutol, and isoniazid. Infections of patients with rapidly growing mycobacteria were treated with clarithromycin in combination with amikacin.

**Table 6:** Treatment regimens for all patients with ATS-defined PNTM, per infecting species, Brazil, 1993–2011*

Species	Treatment regimen (%)†
*Mycobacterium avium* complex	Clarithromycin, amikacin,ethambutol, rifampin (54.7)
	Clarithromycin, ethambutol,quinolone, terizidon (28.3)
	Clarithromycin, amikacin, ethambutol, quinolone‡ (17.0)
*Mycobacterium kansasii*	Rifampin, ethambutol, isoniazid (52.5)
	Rifampin, ethambutol, isoniazid, clarithromycin, amikacin (47.5)
*M. abscessus*	Clarithromycin, amikacin (68.4)
	Clarihromycin, amikacin, doxycycline (10.5)
	Clarithromycin, amikacin, terizidon (21)
*M. massiliense*	Clarithromycin, amikacin (33.3)
	Clarithromycin, amikacin, terizidon (33.3)
	Clarithromycin, amikacin, imipenem, tigeciclin (33.3)
*M. fortuitum*	Clarithromycin, amikacin, quinolone (64.2)
	Clarithromycin, amikacin, terizidon (28.5)
	Clarithromycin, amikacin, quinolone, doxycycline (7.3)

*ATS, AmericanThoracic Society; PNTM, pulmonary nontuberculous mycobacterial disease. †Median duration of treatment, 19 mo. ‡Quinolones: levofloxacin, ciprofloxacin, moxifloxacin, ofloxacin.

## Discussion

We report the emergence of PNTM among patients receiving care at a referral center for MDR TB. Although we were unable to determine the true prevalence of disease caused by these organisms in the state of Rio de Janeiro, the current study confirmed an increasing frequency of patients referred with NTM lung disease. In some industrialized countries, the prevalence of NTM has been found to be increasing (*5*,*17*), although it remains unclear whether this increase is related to increasing awareness of physicians that leads to more frequent diagnosis, to improved laboratory capacity, or to a combination of both factors.

Brazil has a high prevalence of TB, so initial the treatment for TB is based on smear results; culturing is not done and species is not identified before treatment. Thus, patients infected with slowly growing mycobacteria (e.g., *M. kansasii*,) which have a pulmonary manifestation similar to that of *M. tuberculosis* and might respond to empirical treatment with anti-TB drugs, may not have received an appropriate diagnosis. A notable limitation of this study is that we could not accurately determine whether these patients were truly infected with *M. tuberculosis* and had NTM disease as a sequela of their TB disease, or if they were initially infected with NTM. Because empirical treatment is not appropriate for those with *M. tuberculosis* infections, but is done in many parts of the world due to lack of resources, strengthened laboratory capacity is needed to correctly identify the prevalence of emerging NTM disease (*18*). Given the lack of capacity for culture confirmation of all cases of TB, the current disease effects of NTM are likely underestimated in Brazil as well as in other countries with similar resource limitations for mycobacterial diagnostics. The introduction of more affordable rapid diagnostic tools to improve diagnostic capacity should reduce potential misdiagnosis to better estimate the true extent of PNTM.

One third of the NTM patients in this study population were infected with *M. kansasii*, a proportion substantially higher than that seen in the United States (*5*,*19*)and in other areas of Brazil (*10*,*14*) and Australia (*20*). Global geographic variability in *M. kansasii* has been noted; a high prevalence of *M. kansasii* lung disease occurs in Western Europe, Switzerland, and the United Kingdom (*17*,*21*–*23*). In Brazil, available data suggest regional differences in species distribution, although distinct study populations and methods limit comparability of estimates. A prior report from CRPHF, analyzing samples sent from throughout Brazil during 1994–1999, found that of 433 pulmonary isolates, 203 (46.9%) were MAC and 61 (14.0%) were *M. kansasii*. In that study, some regional variability in the relative proportions of *M. kansasii* and MAC was evident, with a somewhat lower proportion of *M. kansasii* in the Southeast (18%) and South regions (13.7%) relative to the North (24.3%) *(**24*). Other studies conducted in different areas of Brazil have found that *M. kansasii* infections range from 16.0% in Bahia (*15*) to 2.2% in São Paulo (*12*) and 0% in the northern Amazonian region (*9*). For *M. kansasii*, municipal water systems are the primary source of *M. kansasii*; thus, the degree of urbanization is likely to affect isolation rates. Climatic and ecologic factors as well as the prevalence of cofactors, such as HIV infection, are also likely to influence the prevalence of NTM species. Brazil encompasses a wide geographic area with large variations in climatic conditions and urbanization. We cannot sort out these factors in our analysis, and future research is needed to address these issues.

*M. kansasii* infection is manifested as a lung disease that is nearly identical to TB with a high prevalence of cavities and a predominance of fibrocavitary lesions in the upper lobe (*3*). However, more recent studies have found a lower frequency of cavitary lesions associated with *M. kansasii* infection in Korea (32%) (*25*) and Israel (57%) (*26*). A culture conversion rate of 95% was found from the study in Korea, with conversion defined as 3 negative samples within a 6-month period (*25*).

In our study, approximately one quarter of patients had no coexisting condition, and 40% had no prior history of TB treatment. NTM have historically been associated with disease in male smokers with COPD (*27*). However, over the last 20 years, the epidemiology has been changing in some industrialized countries, particularly the United States, with most patients identified without known risk factors or with bronchiectasis, all in the context of declining rates of TB (*5*,*6*).

The finding from this study of 1 patient with Marfan syndrome and 1 patient with mitral valve prolapse is consistent with the association of predisposing genetic disorders of the connective tissue with a certain morphotype, as has been found in in the United States (*28*). In our cohort, 8 patients had hepatitis C (from different genotypes and with various degrees of hepatic fibrosis). Of the 8 case-patients with hepatitis C, 5 had *M. kansasii* infection, and the 2 other patients with nonviral cirrhosis of the liver were also infected with *M. kansasii*. Another study found chronic liver disease was an underlying medical problem in patients infected with *M. kansasii* (*26*).

In summary, in Brazil, a country with a high prevalence of TB, the misdiagnosis of NTM disease may lead to inaccurate and inappropriate treatment. This study suggests that despite the high level of TB in Brazil, subpopulations of patients with NTM exist, and these infections are not being adequately detected and treated. Continued monitoring of PNTM is needed in Brazil, and more detailed information should be obtained on pulmonary disease resulting from these infections, including their regional distribution, species, associated coexisting conditions, and treatment response. In addition, adequate laboratory infrastructure and affordable testing are needed at the local level to ensure accurate diagnosis and proper treatment for all.

## References

[1] Falkinham JO III. Epidemiology of infection by nontuberculous mycobacteria. Clin Microbiol Rev. 1996;9:177–215.896403510.1128/cmr.9.2.177PMC172890

[2] Falkinham JO III, Norton CD, LeChevallier MW. Factors influencing number of *Mycobacterial avium, Mycobacterium intracellulare*, and other mycobacteria in drinking water distribution systems. Appl Environ Microbiol. 2001;67:1225–31. 10.1128/AEM.67.3.1225-1231.200111229914PMC92717

[3] Griffith DE, Aksamit T, Brown-Elliott BA, Catanzaro A, Daley C, Gordin F, ATS/IDSA statement: diagnosis, treatment and prevention of nontuberculous mycobacterial diseases. Am J Respir Crit Care Med. 2007;175:367–416. 10.1164/rccm.200604-571ST17277290

[4] Cassidy PM, Hedberg K, Saulson A, McNelly E, Winthrop KL. Nontuberculous mycobacterial disease prevalence and risk factors: a changing epidemiology. Clin Infect Dis. 2009;49:e124–9. 10.1086/64844319911942

[5] Prevots DR, Shaw PA, Strickland D, Jackson LA, Raebel MA, Blosky MA, Nontuberculous mycobacterial lung disease prevalence at four integrated health care delivary systems. Am J Respir Crit Care Med. 2010;182:970–6. 10.1164/rccm.201002-0310OC20538958PMC2970866

[6] Winthrop KL, McNelley E, Kendall B, Marshall-Olson A, Morris C, Cassidy M, Pulmonary nontuberculous mycobacterial disease prevalence and clinical features: an emerging public health disease. Am J Respir Crit Care Med. 2010;182:977–82. 10.1164/rccm.201003-0503OC20508209

[7] Ministry of Health. Brazil. Recommendations for tuberculosis control in Brazil [in Portuguese]. Brasilia (Brazil): The Ministry; 2011.

[8] Oswaldo Cruz Foundation/Ministry of Health. Brazil. MDR TB database–Helio Fraga National TB Reference Center [cited 2013 Jan 16]. http://sitetb.org/

[9] Salem JI, Marôja MF, Carvalho FF, Lima MO, Feuillet A. Mycobacteria other than tubercle bacilli specimens from patients in Manaus (Amazonia, Brasil). Acta Amazon. 1989;19:139–354.

[10] Leite CQF, Viana BHJ, Leite SRA, Juarez E. Incidence of *Mycobacterium tuberculosis* and other mycobacteria on pulmonary infections in Araraquara–SP, 1993. Rev Microbiol, São Paulo. 1995;26:101–5.

[11] Chimara E, Giampaglia CMS, Martins MC, Telles MAS, Ueki SYM, Ferrazoli L. Molecular characterization of *Mycobacterium kansasii* isolates in the State of São Paulo between 1995–1998. Mem Inst Oswaldo Cruz. 2004;99:739–43. 10.1590/S0074-0276200400070001315654431

[12] Pedro HSP, Pereira MIF, Goloni MRA, Ueki SYM, Chimara E. Nontuberculous mycobacteria isolated in São José do Rio Petro, Brazil between 1996 and 2005. J Bras Pneumol. 2008;34:950–5. 10.1590/S1806-3713200800110001019099102

[13] Zamarioli LA, Coellho AGV, Pereira CM, Nascimento ACC, Ueki SYM, Chimara E. Descriptive study of the frequency of nontuberculous mycobacteria in the Baixada Santista region of the state of São Paulo, Brazil. J Bras Pneumol. 2008;34:590–4. 10.1590/S1806-3713200800080000818797743

[14] da Costa AR, Lopes ML, Furlaneto IP, Sousa MS, Lima KVB. Molecular Identification of Nontuberculous mycobacteria isolates in a Brazilian mycobacteria reference laboratory. Diagn Microbiol Infect Dis. 2010;68:390–4. 10.1016/j.diagmicrobio.2010.07.01920884154

[15] Matos ED, Santana MA, Santana MC, Mamede P, Bezerra BL, Panão ED, Nontuberculous mycobacteria at a multiresistant tuberculosis reference center in Bahia: clinical epidemiological Aspects. Braz J Infect Dis. 2004;8:296–304. 10.1590/S1413-8670200400040000515565260

[16] Martins AB, Lemos ACM. Infection with the *Mycobacterium avium* complex in patients without predisposing conditions—a case report and literature review. Braz J Infect Dis. 2005;9:173–9. 10.1590/S1413-8670200500020000916127595

[17] Moore JE, Kruijshaar ME, Ormerod LP, Drobniewski F, Aubakar I. Increasing reports of nontuberculous mycobacteria in England, Wales and Northern Ireland. 1995–2006. BMC Public Health. 2010;10:612. 10.1186/1471-2458-10-61220950421PMC2964631

[18] Guillern M, Usdin M, Arkinstall J. Tuberculosis diagnosis and drug sensitivity testing: an overview of the current diagnostic pipeline. Paris: Médecins Sans Frontières; 2006.

[19] Centers for Disease Control and Prevention. Nontuberculous mycobacteria reported to the Public Health Laboratory Information System by state public health laboratories United States, 1993–1996 [cited 2013 Jan 16]. http://www.cdc.gov/tb/Laboratory_Services/NontuberculousMycobacteria.pdf.

[20] O’Brien DP, Currie BJ, Krause VL. Nontuberculous mycobacterial disease in northern Australia: a case series and review of the literature. Clin Infect Dis. 2000;31:958–68. 10.1086/31813611049777

[21] Debrunner M, Salfinger M, Brandli O, Graevenitz AV. Epidemiology and clinical significance of nontuberculous mycobacteria in patients negative for human immunodeficiency virus in Switzerland. Clin Infect Dis. 1992;15:330–45. 10.1093/clinids/15.2.3301520769

[22] Marras TK, Daley CL. Epidemiology of human pulmonary infection with nontuberculous mycobacteria. Clin Chest Med. 2002;23:553–67. 10.1016/S0272-5231(02)00019-912370992

[23] Evans SA, Colville A, Evans AJ, Crisp AJ, Johnston IDA. Pulmonary *Mycobacterium kansasii* infection: comparison of the clinical features, treatment and outcome with pulmonary tuberculosis. Thorax. 1996;51:1248–52. 10.1136/thx.51.12.12488994524PMC472772

[24] Barreto AMW, Campos CED. Micobactérias “não tuberculosas” no Brasil [Nontuberculous mycobacteria in Brazil]. Boletim de Pneumologia Sanitária. 2000;8:23–32.

[25] Park HK, Koh WJ, Shim TS, Kwon OJ. Clinical vharacteristic and treatment outcomes of *Mycobacterium kansasii* lung disease in Korea. Yonsei Med J. 2010;51:552–6. 10.3349/ymj.2010.51.4.55220499421PMC2880268

[26] Shitrit D, Peled N, Bishara J, Priess P, Pitlik S, Samra Z, Clinical and radiological features of *Mycobacterium kansasii* infection and *Mycobacterium simae* infection. Respi Med. 2008;102:1598–603. 10.1016/j.rmed.2008.05.00418619826

[27] Timpe A, Runyon EH. The relationship of “atypical”acid-fast bacteria to human disease. A preliminary report. J Lab Clin Med. 1954;44:202–9.13184228

[28] Kim RD, Greenberg DE, Ehrmantraut ME, Guide SV, Ding L, Shea Y, Pulmonary nontuberculous mycobacterial disease: prospective study of a distinct preexisting syndrome. Am J Respir Crit Care Med. 2008;178:1066–74. 10.1164/rccm.200805-686OC18703788PMC2720143

# Research article: Clinical and Therapeutic Features of Pulmonary Nontuberculous Mycobacterial Disease, Rio de Janeiro, Brazil

**Authors:** Karla Gripp Couto de Mello, Fernanda C. Queiroz Mello, Liamar Borga, Valeria Rolla, Rafael S. Duarte, Elizabeth P. Sampaio, Steven M. Holland, D. Rebecca Prevots, Margareth P. Dalcolmo

**Affiliations:** Author affiliations: National School of Public Heath, Oswaldo Cruz Foundation, Rio de Janeiro, Brazil (K.G. Couto de Mello, L. Borga, M.P. Dalcolmo);; School of Medicine, Federal University of Rio de Janeiro, Rio de Janeiro (F.C. Queiroz Mello);; Oswaldo Cruz Institute, Rio de Janeiro (V. Rolla, E.P. Sampaio);; Federal University of Rio de Janeiro (R.S. Duarte);; National Institutes of Health, Bethesda, Maryland, USA (S.M. Holland, D.R. Prevots)

## Medscape CME Quiz

## Earning CME Credit

To obtain credit, you should first read the journal article. After reading the article, you should be able to answer the following, related, multiple-choice questions. To complete the questions (with a minimum 70% passing score) and earn continuing medical education (CME) credit, please go to www.medscape.org/journal/eid. Credit cannot be obtained for tests completed on paper, although you may use the worksheet below to keep a record of your answers. You must be a registered user on Medscape.org. If you are not registered on Medscape.org, please click on the New Users: Free Registration link on the left hand side of the website to register. Only one answer is correct for each question. Once you successfully answer all post-test questions you will be able to view and/or print your certificate. For questions regarding the content of this activity, contact the accredited provider, CME@medscape.net. For technical assistance, contact CME@webmd.net. American Medical Association’s Physician’s Recognition Award (AMA PRA) credits are accepted in the US as evidence of participation in CME activities. For further information on this award, please refer to http://www.ama-assn.org/ama/pub/category/2922.html. The AMA has determined that physicians not licensed in the US who participate in this CME activity are eligible for *AMA PRA Category 1 Credits*™. Through agreements that the AMA has made with agencies in some countries, AMA PRA credit may be acceptable as evidence of participation in CME activities. If you are not licensed in the US, please complete the questions online, print the certificate and present it to your national medical association for review.

## Article Title:  Clinical and Therapeutic Features of Pulmonary Nontuberculous Mycobacterial Disease, Rio de Janeiro, Brazil, 1993–2011

## CME Questions

**1. You are seeing a 55-year-old man with a 4-month history of cough, night sweats, and weight loss. You suspect that he has pulmonary tuberculosis, but you also consider whether he has pulmonary nontuberculous mycobacterial (PNTM) disease. What should you consider regarding PNTM disease?**

A. Immunosuppression affects the risk of pulmonary tuberculosis but not PNTM disease

B. The highest prevalence of PNTM disease in the United States is among men between the ages of 20 and 35 years

C. The prevalence of PNTM disease is higher in the United States compared with Brazil

D. Pulmonary infection with *Mycobacterium kansasii* has a clinical presentation very similar to that of pulmonary tuberculosis

**2. Which of the following statements regarding the clinical presentation of PNTM disease among patients in the current study is most accurate?**

A. Nearly all patients had significant comorbid illnesses

B. Most patients reported a history of smoking

C. Systemic symptoms were more common than respiratory symptoms

D. Less than 10% of patients had received prior treatment for tuberculosis

**3. You order sputum cultures for this patient. Which of the following organisms were most common in cases of PNTM disease in the current study?**

A. *M. abscessus* and *M. fortuitum*

B. *M. abscessus* and *M. avium* complex (MAC)

C. *M. kansasii* and MAC

D. *M. kansasii* and *M. fortuitum*

**4. On further testing, the patient does appear to have PNTM disease. Which of the following organisms was associated with the LOWEST cure rate in the current study?**

A. *M. fortuitum*

B. *M. abscessus*

C. MAC

D. *M. kansasii*

## Activity Evaluation

**Table:** 

**1. The activity supported the learning objectives.**				
Strongly Disagree				Strongly Agree
1	2	3	4	5
**2. The material was organized clearly for learning to occur.**				
Strongly Disagree				Strongly Agree
1	2	3	4	5
**3. The content learned from this activity will impact my practice.**				
Strongly Disagree				Strongly Agree
1	2	3	4	5
**4. The activity was presented objectively and free of commercial bias.**				
Strongly Disagree				Strongly Agree
1	2	3	4	5
